# Sexuality and Gender Diversity Among Adolescents in Australia, 2019-2021

**DOI:** 10.1001/jamanetworkopen.2024.44187

**Published:** 2024-10-28

**Authors:** Jennifer L. Marino, Aliza Werner-Seidler, Kate Maston, Ashleigh Lin, Yael Perry, Sarita Bista, Cristyn Davies, Helen Christensen, S. Rachel Skinner

**Affiliations:** 1 Specialty of Child and Adolescent Health, Faculty of Medicine and Health, The University of Sydney, Westmead, New South Wales, Australia; 2 Centre for Adolescent Health, Murdoch Children’s Research Institute, Parkville, Victoria, Australia; 3 Centre for Epidemiology and Biostatistics, School of Population and Global Health, University of Melbourne, Parkville, Victoria, Australia; 4 Black Dog Institute, University of New South Wales, Sydney, New South Wales, Australia; 5 School of Population and Global Health, University of Western Australia, Perth, Western Australia, Australia; 6 The Kids Research Institute, University of Western Australia, Perth, Western Australia, Australia; 7 The Children’s Hospital at Westmead, Westmead, New South Wales, Australia; 8 School of Social Sciences, Western Sydney University, New South Wales, Australia; 9 Discipline of Psychiatry and Mental Health, University of New South Wales, Sydney, New South Wales, Australia

## Abstract

**Importance:**

Sexuality- and gender-diverse (SGD) young people experience substantial health disparities relative to cisgender heterosexual peers. Little is known about SGD adolescents younger than 15 years.

**Objective:**

To describe SGD prevalence and associated factors in a population-representative cohort of younger adolescents in Australia.

**Design, Setting, and Participants:**

This prospective cohort study was part of the Future Proofing Study, with enrollment of year 8 students at 134 Australian secondary schools from 2019 to 2021 and annual follow-ups for 5 years. Data were analyzed from June 20, 2023, to June 6, 2024.

**Main Outcome and Measures:**

Outcomes of interest were baseline self-reported gender and sexuality identities, individual characteristics, and mental health and disability diagnoses, as well as school characteristics.

**Results:**

Among 6388 participants, median (IQR) age was 13.9 (13.6-15.8), with a range of 10.7 to 17.5 years. Most participants attended school in a major city (76.0%), were born in Australia (91.4%), and spoke English at home (93.7%). Approximately half (3122 participants; 48.9% [95% CI, 45.2%-59.0%]) identified as female or girls, and 46.5% (2973 participants; 95% CI, 39.8%-53.4%) identified as male or boys. The overall proportion of transgender identity was 3.3% (95% CI, 2.7%-3.9%), with 23 participants (0.4%) identifying as transgender boys, 10 participants (0.2%) identifying as transgender girls, 117 participants (1.8%) identifying as transgender nonbinary, and 59 participants (0.9%) identifying as another transgender identity. The overall proportion of sexuality diversity was 12.0% (95% CI, 10.4%-13.8%). The proportion of cisgender participants who were sexuality-diverse (13.0% [95% CI, 11.4%-14.8%] of girls and 4.7% [95% CI, 3.7%-5.9%] of boys) was lower than the proportion among gender-diverse participants, which ranged from 30.0% (95% CI, 9.3%-64.1%) of transgender girls to 91.5% (95% CI, 81.3%-96.4%) of those with another diverse gender identity. Gender diversity and sexuality diversity were strongly associated (odds ratio [OR], 66.24; 95% CI, 38.23-114.80), and both were negatively associated with age (gender diversity: OR per 1-year older, 0.61; 95% CI, 0.49-0.76; sexuality diversity: OR per 1-year older, 0.78; 95% CI, 0.65-0.93) and positively with mental health diagnosis (gender diversity: OR, 2.41; 95% CI, 1.79-3.24; sexuality diversity: OR, 2.50; 95% CI, 2.10-2.98), and disability diagnosis (gender diversity: OR, 2.39; 95% CI, 1.68-3.40; sexuality diversity: OR, 1.96; 95% CI, 1.64-2.36). While there were significant associations between individual and school characteristics and responses to gender and sexuality identity items, patterns of association differed, with no consistent association with economic disadvantage.

**Conclusions and Relevance:**

This cohort study of young adolescents found higher rates of SGD than among samples of older adolescents. The significant associations with younger age, poorer mental health, and disability underscored the urgent need for inclusive programs to promote a safe and welcoming environment in schools, health care settings, and communities.

## Key Points

**Question:**

What was the prevalence of sexuality and gender diversity among young adolescents in Australia during 2019 to 2021?

**Findings:**

In this cohort study of 6388 students (mean age, 13.9 years), 3.3% were gender diverse and 12.0% were sexuality diverse. Sexuality and gender diversity were associated with each other, and both were associated with younger age and with diagnoses of poor mental health and disability.

**Meaning:**

This study found that it was more common for younger adolescents to report being sexuality or gender diverse and more common for these adolescents to report mental health difficulties and disabilities.

## Teaser

This cohort study explores the prevalence of and factors associated with sexuality and gender diversity in a population-representative cohort of younger adolescents in Australia from 2019 to 2021.

## Introduction

Sexuality- and gender-diverse (SGD) young people experience substantial health disparities relative to cisgender heterosexual peers, including higher rates of sexually transmitted infections,^1^ unplanned pregnancy,^2,3,4^ and mental illness.^5,6,7,8,9^ These disproportionate morbidities are thought to arise primarily from minority stress, caused by the social, psychological, and structural discrimination and exclusion experienced by stigmatized minority groups, including SGD individuals. While some population-based data exist about adolescents aged older than 14 years,^10,11,12^ little is known about younger adolescents, particularly with respect to gender diversity. Recent surveys report a higher proportion of adolescents than adults are SGD^13,14,15,16,17^, suggesting a secular trend or a greater willingness to self-report diverse identities.

There is also evidence that uncertainty or unwillingness to report sexuality and gender identities is associated with adverse outcomes, including substance use,^18,19,20,21,22,23,24^ violence and victimization,^10,12,19,20,25,26,27^ and health problems.^12,20,21,22,28,29,30^ Yet many studies exclude “I don’t know” and “I am not sure” responses from analyses, considering them refusals. However, an unwillingness to disclose may represent a lack of clarity, support, or safety. Thus, we sought to describe prevalence and correlates of SGD, preferring not to report, and unsure responses among a new population-representative cohort of year-8 students in Australia.^31^

## Methods

This cohort study was approved by the University of New South Wales Human Research Ethics Committee, the State Education Research Applications Process for the New South Wales Department of Education, and relevant Catholic Schools Dioceses. For all participants, their parents provided informed consent, and participants further provided informed consent. This report follows the Strengthening the Reporting of Observational Studies in Epidemiology (STROBE) reporting guideline.

The Future Proofing study is a prospective cohort study of youth mental health that enrolled 3 Australian year 8 cohorts (typically aged 13-14 years) in August to September 2019, August to November 2020, and April to December 2021.^31,32^ All 1036 government and independent secondary schools in New South Wales, eligible New South Wales Catholic schools, and independent secondary schools in capital cities were invited to participate. Of 200 schools consenting to participate, 66 withdrew because of COVID-19 lockdowns. In the 134 participating schools, 20 533 students were invited to participate. Written consent was obtained from 7577 parents, and baseline data were collected from 6388 students. Participants also provided written personal consent and completed questionnaires online in schools during class time, using their computer, smartphone, or tablet.

School characteristics ascertained from the Australian Curriculum, Assessment and Reporting Authority database included locational remoteness, sector, and index of community socioeducational advantage (ICSEA,^33,34^ ranging from 500-1300, with a population median of 1000 and SD of 100; higher values indicate higher advantage.)

Individual characteristics were self-reported, including demographics (country of birth, language spoken at home, perceived family socioeconomic status, family structure), mental health and disability diagnoses, sex recorded at birth, gender (a mandatory and unskippable item), sexuality, and sexual attraction (eBox 1 in Supplement 1). For purposes of these analyses, SGD includes sexual orientation (identity, attraction, and behavior), gender identity, and gender expression.

### Statistical Analysis

Analyses were conducted in Stata version 15.0 (StataCorp) from June 20, 2023, to June 6, 2024. Categorical variables were summarized with counts and proportions; and continuous variables with medians, IQRs, and ranges. For proportions, 95% CIs were calculated using sandwich estimators to account for clustering by school. Proportions of sexuality identities by gender diversity (gender diverse and cisgender) and gender (male and female) were compared using multinomial logistic regression. All regression models were adjusted for clustering by school. All hypothesis tests were 2-sided, with statistical significance defined as *P* < .05 for tests; for measures of association, significance was determined as 95% CIs excluding the null. We did not adjust *P* values for multiple comparisons.

As some groups were small, sex recorded at birth, gender identity, and sexuality identity were regrouped for further analyses. Sex recorded at birth was regrouped into categories male, female, and unsure or prefer not to report. Gender identity was regrouped into categories of cisgender, gender-diverse, prefer not to report, and missing or uninterpretable.^35,36^ The gender-diverse category included those who recorded male at birth who reported female gender, those recorded female at birth who reported male gender, and all who reported nonbinary or another gender. Sexuality identity was regrouped into categories heterosexual, sexuality diverse, prefer not to report, unsure, and missing or uninterpretable. Sexuality diversity included those who reported gay or lesbian, bisexual, pansexual, asexual, or another term.

Associations between individual and school characteristics and regrouped categories were examined using multinomial logistic regression, with cisgender as the reference category for gender identity and heterosexual as the reference group for sexual identity. To build each multivariable model, we first included all individual and school characteristics significantly associated with each identity at either the category level (by 95% CI inspection and simple postestimation Wald test) or overall across the variable (by composite postestimation Wald test) then eliminated those variables that did not remain significantly associated at the category or overall level. The exception was the association between gender diversity and sexuality diversity, which, for interpretability, was excluded from the final multivariable models.

## Results

Among a total of 6388 participants, the median (IQR) age was 13.9 (13.6-15.8) years (range, 10.7-17.5 years). Participant characteristics have been previously published^31^; for this report, we generated 95% CIs, which are presented in eTable 1 in Supplement 1. Briefly, most participants attended school in a major city (76.0%), were born in Australia (91.4%), spoke English at home (93.7%) and lived in a household with 2 biological parents (78.4%). Approximately one-half of participants attended government schools (50.9%). Most participants (70.5%) attended a school with higher-than-average ICSEA. Mental health diagnoses were reported by 17.7% of participants, most commonly generalized anxiety disorder (7.8%), social anxiety disorder (6.0%), and attention-deficit hyperactivity disorder (6.2%). Disability diagnoses were reported by 12.5% of participants, most commonly visual impairment (5.9%), autism spectrum disorders (2.7%), and specific learning disability (2.1%).

### Sex Recorded at Birth

Of 6388 participants, 3329 (52.1% [95% CI, 45.2%-59.0%]) were recorded female at birth, 2970 (46.5% [95% CI, 39.8%-53.4%]) were recorded male at birth, 33 (0.5% [95% CI, 0.3%-0.8%]) were unsure, 52 (0.8% [95% CI, 0.6%-1.2%]) preferred not to report, and 4 (0.06% [95% CI, 0.02%-0.2%]) had missing or uninterpretable data.

### Gender Identity

Current gender by sex recorded at birth is reported in Table 1. Overall, 3329 participants (48.9%) identified as female (girls), and 2970 participants (46.5%) identified as male (boys). Most participants reported gender congruent with sex recorded at birth (3101 of 3329 participants who were recorded female at birth [93.2%] identified as female; 2918 of 2970 participants who were recorded male at birth [98.2%] identified as male), with an overall prevalence of cisgender identity of 94.2% (6019 participants [95% CI, 93.4%-95.0%]). Many participants who were unsure, preferred not to report, or were missing sex recorded at birth chose to report a current gender (61 of 89 participants [68.5%; 95% CI, 56.8%-78.3%]), but within this group, transgender status could be confirmed only for those who reported nonbinary (13 participants) or another (5 participants) gender identity, who were counted as gender-diverse. There were 109 participants (1.7% [95% CI, 1.3%-2.2%]) who preferred not to report gender identity.

**Table 1.** Current Gender by Sex Recorded at Birth

Current gender	Sex recorded at birth, No. (%) [95% CI]					
	All (N = 6388)	Female (n = 3329)	Male (n = 2970)	Unsure (n = 33)	Prefer not to report (n = 52)	Missing or uninterpretable (n = 4)^a^
Female (girl)	3122 (48.9) [45.2-59.0]	3101 (93.2) [91.8-94.3]	10 (0.3) [0.2-0.6]	4 (12.1) [4.5-29.0]	7 (13.5) [6.3-26.4]	0
Male (boy)	2973 (46.5) [39.8-53.4]	23 (0.7) [0.5-1.0]	2918 (98.2) [97.6-98.7]	17 (51.5) [33.8-68.9]	14 (26.9) [16.2-41.1]	1 (25.0) [6.2-62.8]
Nonbinary	117 (1.8) [1.5-2.3]	91 (2.7) [2.1-3.5]	13 (0.4) [0.3-0.8]	3 (9.1) [2.9-25.2]	9 (17.3) [8.8-31.2]	1 (25.0) [2.7-79.9]
Another identity	59 (0.9) [0.6-1.3]	52 (1.6) [1.1-2.2]	2 (0.07) [0.02-0.03]	2 (6.1) [1.7-19.0]	3 (5.8) [1.9-15.9]	0
Prefer not to report	109 (1.7) [1.3-2.2]	62 (1.9) [1.4-2.5]	23 (0.8) [0.5-1.2]	5 (15.2) [6.4-31.7]	18 (34.6) [20.6-51.9]	1 (25.0) [6.2-62.8]
Uninterpretable^b^	8 (0.1) [0.07-0.2]	0	4 (0.1) [0.05-0.4]	2 (6.1) [1.5-21.4]	1 (1.9) [2.7-12.6]	1 (25.0) [2.7-79.9]

^a^ Uninterpretable responses included “attack helicopter,”^35,36^ “German attack helicopter,” and “none.”

^b^ Uninterpretable responses included “attack helicopter,” “an Alpha Male German Attack Helicopter,” “Lamborghini tractor,” “alien for sure,” “level 13 ice wizard from coc,” “microwave,” and “none.”

The prevalence of transgender male identity was 0.4% (95% CI, 0.2%-0.5%; 23 participants); transgender female identity, 0.2% (95% CI, 0.1%-0.3%; 10 participants); nonbinary identity, 1.8% (95% CI, 1.5%-2.3%; 117 participants); and other gender identities, 0.9% (95% CI, 0.7%-1.3%; 59 participants), with an overall prevalence of any transgender identity of 3.3% (95% CI, 2.7%-3.9%; 209 participants). Participants used diverse terms to describe other transgender identities (eBox 2 in Supplement 1), most commonly “genderfluid” (18 participants), “demi-girl” (7 participants), and “agender” (5 participants). Nine participants identified themselves with combinations of pronouns or specified using all pronouns, and 5 participants described not knowing or being unsure.

### Sexuality Identity

The overall proportion of sexuality diversity was 12.0%, with 8.8% unsure, 4.6% preferring not to report, 4.3% missing, and 0.3% uninterpretable responses (Table 2). The proportion of cisgender participants who were sexuality diverse (13.0% of females, 4.7% of males) was substantially lower than among gender-diverse participants, which ranged from 30.0% among transgender females to 91.5% of those with another transgender identity. Sexuality identity differed significantly by gender identity, with 539 of 6019 of cisgender participants (9.0% [95% CI, 7.8%-10.3%]) reporting sexuality diversity, in contrast to 162 of 209 gender-diverse participants (77.5% [95% CI, 69.9%-83.6%]), 62 of 109 participants who preferred not to report gender identity (56.9% [95% CI, 46.8%-66.4%]), and 4 of 51 participants who did not respond to the gender identity item (7.8% [95% CI, 3.0%-19.0%]) (overall *P* < .001). The overall proportion who identified as gay or lesbian was 1.6%; bisexual, 6.5%; pansexual, 1.9%; and asexual, 1.0%. Gender-diverse participants were significantly more likely than cisgender participants to identify as gay or lesbian (28 participants [13.4%] vs 51 participants [1.0%]; *P* < .001), bisexual (43 participants [20.6%] vs 348 participants [5.8%]; *P* < .001), pansexual (48 participants [23.0%] vs 61 participants [1.1%]; *P* < .001), asexual (14 participants [6.7%] vs 45 participants [0.8%]; *P* < .001), or another sexuality identity (29 participants [14.0%] vs 26 participants [0.4%]; *P* < .001). Girls were significantly more likely than boys to identify as bisexual (280 girls [9.0%] vs 73 boys [2.4%]; *P* < .001) but not gay or lesbian (40 girls [1.3%] vs 23 boys [0.8%]; *P* = .08), pansexual (42 girls [1.3%] vs 24 boys [0.8%]; *P* = .07), asexual (27 girls [0.9%] vs 21 boys [0.7%]; *P* = .54), or another sexuality identity (19 girls [0.6%] vs 13 boys [0.4%]; *P* = .35).

**Table 2.** Sexuality Identity and Sexual Attraction by Gender Identity

Measure	All (N = 6388)	Gender identity, No. (%) [95% CI]							
		Cisgender		Transgender			Another identity (n = 59)	Prefer not to report (n = 109)	Missing or unclassifiable (n = 51)^a^
		Girl (n = 3101)	Boy (n = 2918)	Girl (n = 10)	Boy (n = 23)	Nonbinary (n = 117)			
**Sexuality identity**									
Heterosexual	4472 (70.0) [67.5-72.4]	2134 (68.8) [66.3-71.2]	2274 (77.9) [75.5-80.2]	5 (50.0) [24.5-75.4]	4 (17.4) [6.5-38.8]	11 (9.4) [4.4-18.9]	0	12 (11.0) [6.1-19.0]	32 (62.7) [48.8-74.9]
Sexuality diverse	767 (12.0) [10.4-13.8]	403 (13.0) [11.4-14.8]	136 (4.7) [3.7-5.9]	3 (30.0) [9.3-64.1]	16 (69.6) [47.8-85.1]	89 (76.1) [65.9-83.9]	54 (91.5) [81.3-96.4]	62 (56.9) [46.8-66.4]	4 (7.8) [3.0-19.0]
Gay or lesbian	103 (1.6) [1.3-2.1]	39 (1.3) [0.9-1.7]	20 (0.7) [0.4-1.7]	1 (10.0) [1.3-48.0]	2 (8.7) [2.1-29.4]	15 (12.8) [8.1-19.6]	10 (16.9) [9.7-28.1]	15 (13.8) [8.6-21.3]	1 (2.0) [0.3-12.7]
Bisexual	414 (6.5) [5.6-7.6]	279 (9.0) [7.9-10.3]	69 (2.4) [1.7-3.2]	1 (10.0) [1.3-48.0]	4 (17.4) [5.4-43.5]	26 (22.2) [14.8-31.9]	12 (20.3) [12.5-31.4]	23 (21.1) [14.5-29.7]	0
Pansexual	119 (1.9) [1.5-2.3]	41 (1.3) [0.9-1.9]	20 (0.7) [0.4-1.1]	0	4 (17.4) [6.5-38.8]	28 (23.9) [16.9-32.7]	16 (27.1) [17.3-39.8]	9 (8.3) [4.70-14.2]	1 (2.0) [0.3-13.2]
Asexual	67 (1.0) [0.8-1.4]	26 (0.8) [0.5-1.3]	19 (0.7) [0.4-1.1]	0	1 (4.3) [5.9-25.8]	8 (6.8) [3.7-12.4]	5 (8.5) [3.7-18.2]	6 (5.5) [2.6-11.4]	2 (3.9) [1.0-14.2]
Another identity^b^	64 (1.0) [0.8-1.3]	18 (0.6) [0.3-1.0]	8 (0.3) [0.1-0.5]	1 (10.0) [1.3-48.0]	5 (21.7) [9.1-43.4]	12 (10.3) [6.2-16.6]	11 (18.6) [10.5-31.0]	9 (8.3) [4.2-15.6]	0
Unsure	559 (8.8) [7.8-9.8]	325 (10.5) [9.1-12.0]	202 (6.9) [5.8-8.2]	1 (10.0) [1.3-48.0]	1 (4.3) [0.6-25.8]	7 (6.0) [2.6-13.0]	3 (5.1) [1.6-14.6]	14 (12.8) [8.0-20.0]	6 (11.8) [5.4-23.7]
Prefer not to report	296 (4.6) [4.0-5.3]	138 (4.5) [3.7-5.3]	133 (4.6) [3.8-5.5]	1 (10.0) [1.7-41.9]	1 (4.3) [0.6-25.8]	4 (3.4) [1.3-8.4]	0	15 (13.8) [9.2-20.1]	4 (7.8) [3.1-18.6]
Missing	275 (4.3) [3.3-5.5]	98 (3.2) [2.3-4.3]	158 (5.4) [4.0-7.3]	0	1 (4.3) [0.6-25.8]	6 (5.1) [2.3-11.1]	2 (3.4) [0.8-12.7]	6 (5.5) [2.5-11.5]	4 (7.8) [3.1-18.3]
Uninterpretable^c^	19 (0.3) [0.2-0.5]	3 (0.01) [0.03-0.3]	15 (0.5) [0.3-0.9]	0	0	0	0	0	1 (2.0) [0.3-12.3]
**Sexual attraction**									
Only females	2376 (37.2) [31.6-43.1]	31 (1.0) [0.7-1.4]	2257 (77.3) [74.9-79.6]	5 (50.0) [24.5-75.4]	2 (8.7) [2.1-29.4]	24 (20.5) [13.5-29.9]	9 (15.3) [7.8-27.8]	22 (20.2) [13.2-29.7]	26 (51.0) [36.0-65.8]
Mostly females	329 (5.2) [4.5-5.9]	96 (3.1) [2.4-3.9]	170 (5.8) [4.8-7.0]	0	3 (13.0) [4.4-32.7]	27 (23.1) [15.9-32.2]	12 (20.3) [12.6-31.1]	18 (16.5) [10.8-24.5]	3 (5.9) [1.8-17.5]
Equally male and female	278 (4.4) [3.7-5.1]	173 (5.6) [4.7-6.6]	53 (1.8) [1.4-2.4]	0	3 (13.0) [4.2-34.1]	23 (19.7) [13.2-28.3]	11 (18.6) [11.0-29.8]	13 (11.9) [7.0-19.6]	2 (3.9) [0.9-15.0]
Mostly males	411 (6.4) [5.5-7.6]	358 (11.5) [10.2-13.0]	22 (0.8) [0.5-1.2]	1 (10.0) [1.3-48.0]	3 (13.0) [4.2-34.1]	7 (6.0) [3.0-11.7]	3 (5.1) [1.7-14.3]	14 (12.8) [7.7-20.6]	3 (5.9) [1.8-17.4]
Only males	1980 (31.0) [26.6-35.7]	1948 (62.8) [59.8-65.8]	15 (0.5) [0.3-0.9]	2 (20.0) [4.8-55.5]	4 (17.4) [6.5-38.8]	1 (0.9) [0.1-5.8]	2 (3.4) [0.8-13.0]	3 (2.8) [0.9-8.0]	5 (9.8) [3.6-24.3]
Other	90 (1.4) [1.1-1.8]	27 (0.9) [0.6-1.3]	23 (0.8) [0.5-1.1]	0	1 (4.3) [0.6-25.8]	19 (16.2) [10.3-24.7]	11 (18.6) [10.3-31.4]	9 (8.3) [4.7-14.2]	0
Unsure	404 (6.3) [5.5-7.2]	241 (7.8) [6.7-9.0]	127 (4.4) [3.5-5.4]	1 (10.0) [1.3-48.0]	4 (17.4) [6.9-37.4]	8 (6.8) [3.5-13.0]	9 (15.3) [8.3-26.3]	10 (9.2) [4.8-17.0]	4 (7.8) [2.4-22.6]
Prefer not to report	259 (4.1) [3.4-4.8]	131 (4.2) [3.5-5.1]	101 (3.5) [2.7-4.5]	1 (10.0) [1.7-41.9]	2 (8.7) [2.1-29.4]	3 (2.6) [0.9-7.4]	0	16 (14.7) [9.2-22.6]	5 (9.8) [4.2-21.2]
Missing	261 (4.1) [3.1-5.3]	96 (3.1) [2.3-4.2]	150 (5.1) [3.8-7.0]	0	1 (4.3) [0.6-25.8]	5 (4.3) [1.8-9.9]	2 (3.4) [0.8-12.7]	4 (3.7) [1.4-9.3]	3 (5.9) [2.0-16.1]

^a^ Missingness differs from that in Table 1 because gender identity was derived from sex recorded at birth (which could be unsure, prefer not to report, or missing) and current gender.

^b^ See eBox 2 in Supplement 1 for specific identities.

^c^ Uninterpretable responses included “Walmart bag,” “turtle,” “curry puff,” “fire truck,” “im atracted to attack helecopter and my girlfrend” (sic), “nahhh,” “Christian,” “healthy person,” “default,” “default settings,” “y,” “none,” “no,” “attack helicopter,” “normal,” and “male.”

Among 31 free-text responses, participants most commonly provided more than 1 term (eg, “pansexual/asexual”) or a detailed explanation (eBox 2 in Supplement 1). Eight participants specified they were omnisexual. Eleven participants provided a combination of terms describing sexuality and romantic identity.

### Sexual Attraction

Sexual attraction by gender identity is reported in Table 2. Close to two-thirds of cisgender girls were exclusively male-attracted (62.8%); 1.0%, exclusively female-attracted; 20.2%, attracted to males and females; 0.9%, other; 7.8%, unsure; 4.2%, preferred not to report; and 3.1%, did not respond. More than three-quarters of cisgender boys were exclusively female-attracted (77.3%); 0.5%, exclusively male-attracted; 9.0%, attracted to males and females; 0.8%, other; 4.4%, unsure; 3.5%, preferred not to report; and 5.1%, did not respond. Among transgender boys and girls, close to one-third (9 of 33 participants [27.3%]) were exclusively attracted to the same gender, whereas among nonbinary participants, more than one-fifth (20.5%) were exclusively attracted to females, but only 0.9% were exclusively attracted to males. No transgender girls and only 1 transgender boy reported other attraction, similar to cisgender participants, while 16.2% of nonbinary participants and 18.6% of participants of another gender identity reported other attraction.

### Individual and School Characteristics by Gender Identity

Gender diversity and sexuality diversity were strongly associated (odds ratio [OR], 66.24; 95% CI, 38.23-114.80). Before and after adjustment for individual and school characteristics, compared with cisgender status, gender diversity was negatively associated with age (adjusted odds ratio [aOR] per 1 year, 0.59 [95% CI, 0.47-0.74) and positively associated attending a government school (aOR, 1.74 [95% CI, 1.20-2.54]), having a mental health diagnosis (aOR, 2.07 [95% CI, 1.50-2.84)], and having a disability diagnosis (aOR, 2.10 [95% CI, 1.45-3.03]) (Table 3). Although this could not be explored in multivariable modeling because of low power and collinearity, gender diversity was positively associated with the 3 most common mental health diagnoses in the cohort (generalized anxiety disorder, social phobia, and attention-deficit hyperactivity disorder), and with 2 of the 3 most common disability diagnoses (visual impairment and autism spectrum disorders) (Table 3). Associations of gender diversity with lower school socioeducational advantage and family structure were not maintained in the model adjusted for other variables.

**Table 3.** Unadjusted (Bivariate) and Adjusted (Multivariate) Multinomial Regression of Gender Diversity on Individual and School Characteristics

Variable	Cisgender, No. (%) (n = 6019)^a^	Gender-diverse (n = 209)		Prefer not to report (n = 109)		Missing (n = 51)		Overall *P* value^b^	aOR (95% CI)^a^			Overall *P* value^b^
		No. (%)	OR (95% CI)	No. (%)	OR (95% CI)	No. (%)	OR (95% CI)		Gender-diverse (n = 209)	Prefer not to report (n = 109)	Missing (n = 51)	
Age, median (IQR) [range], y	13.9 (13.6-14.3) [10.7-17.5]	13.8 (13.4-14.1) [11.5-16.3]	0.61 (0.49-0.76)	13.9 (13.6-14.2) [12.6-16.1]	0.98 (0.70-1.37)	13.8 (13.5-14.5) (12.5-16.5)	1.19 (0.67-2.10)	<.001	0.59 (0.47-0.74)	0.97 (0.69-1.34)	1.17 (0.55-2.06)	<.001
School type												
Government	3030 (50.3)	135 (64.6)	1.80 (1.26-2.57)	65 (55.6)	1.30 (0.77-2.19)	25 (49.0)	0.95 (0.50-1.79)	.01	1.74 (1.20-2.54)	1.47 (0.85-2.52)	0.90 (0.45-1.78)	.03
Nongovernment	2989 (49.7)	75 (35.4)	1 [Reference]	52 (44.4)	1 [Reference]	26 (51.0)	1 [Reference]		1 [Reference]	1 [Reference]	1 [Reference]	
School ICSEA												
1000 or less	1764 (29.3)	81 (38.8)	1.53 (1.07-2.19)	31 (26.5)	0.92 (0.52-1.61)	12 (23.5)	0.74 (0.37-1.48)	.09	NA^c^	NA^c^	NA^c^	NA^c^
1001 or greater	4255 (70.7)	128 (61.2)	1 [Reference]	86 (73.5)	1 [Reference]	39 (76.5)	1 [Reference]		NA^c^	NA^c^	NA^c^	
Location												
Major cities	4573 (76.0)	150 (71.8)	0.80 (0.54-1.19)	100 (85.5)	1.84 (1.03-3.28)	23 (23.5)	1.03 (0.50-2.10)	.12	0.96 (0.66-1.40)	2.08 (1.10-3.95)	1.04 (0.48-2.24)	.15
Regional	1446 (24.0)	59 (28.2)	1 [Reference]	17 (14.5)	1 [Reference]	39 (76.5)	1 [Reference]		1 [Reference]	1 [Reference]	1 [Reference]	
Country of birth												
Australia	5514 (91.6)	190 (90.9)	1 [Reference]	100 (85.5)	1 [Reference]	44 (86.3)	1 [Reference]	.15	NA^c^	NA^c^	NA^c^	NA^c^
Other	404 (8.4)	19 (9.1)	1.09 (0.70-1.71)	17 (14.5)	1.74 (0.95-3.19)	7 (13.7)	1.74 (0.73-4.14)		NA^c^	NA^c^	NA^c^	
Language spoken at home												
English	5645 (93.8)	192 (91.9)	1 [Reference]	105 (89.7)	1 [Reference]	46 (90.2)	1 [Reference]	.55	NA^c^	NA^c^	NA^c^	NA^c^
Other	373 (6.2)	17 (8.1)	1.34 (0.73-2.47)	12 (10.3)	1.53 (0.70-3.32)	5 (9.8)	1.65 (0.54-4.99)		NA^c^	NA^c^	NA^c^	
Perceived SES												
Medium/high	4693 (78.0)	149 (71.3)	1 [Reference]	80 (68.4)	1 [Reference]	33 (64.7)	1 [Reference]	<.001	1 [Reference]	1 [Reference]	1 [Reference]	.001
Low	491 (8.2)	24 (11.5)	1.54 (1.00-2.38)	8 (6.8)	0.90 (0.36-2.30)	7 (13.7)	2.03 (0.96-4.26)		1.20 (0.77-1.87)	0.82 (0.33-2.04)	2.01 (0.91-4.43)	
Prefer not to report	835 (13.9)	36 (17.2)	1.36 (0.84-2.19)	29 (24.8)	2.13 (1.39-3.26)	11 (21.6)	1.87 (1.00-3.52)		1.23 (0.76-2.00)	2.04 (1.32-3.15)	1.93 (1.03-3.61)	
Family structure												
2 Parents	4756 (79.0)	142 (67.9)	1 [Reference]	82 (70.1)	1 [Reference]	35 (68.6)	1 [Reference]	<.001	NA^c^	NA^c^	NA^c^	NA^c^
Blended/stepfamily	593 (9.9)	32 (15.3)	1.81 (1.23-2.66)	16 (13.7)	1.58 (0.96-2.62)	8 (15.7)	1.83 (0.87-3.87)		NA^c^	NA^c^	NA^c^	
Single parent	616 (10.2)	30 (14.4)	1.63 (1.06-2.51)	16 (13.7)	1.52 (0.80-2.92)	8 (15.7)	1.76 (0.77-4.03)		NA^c^	NA^c^	NA^c^	
Other, no family	54 (0.9)	5 (2.4)	3.10 (1.17-8.24)	3 (2.6)	3.48 (1.10-11.0)	0	-		NA^c^	NA^c^	NA^c^	
Mental health diagnosis												
None	4996 (83.0)	140 (67.0)	1 [Reference]	85 (72.6)	1 [Reference]	40 (78.4)	1 [Reference]	NA	1 [Reference]	1 [Reference]	1 [Reference]	NA
Any	1023 (17.0)	69 (33.0)	2.41 (1.79-3.24)	32 (27.4)	1.77 (1.23-2.54)	11 (21.6)	1.34 (0.75-2.40)	<.001	2.07 (1.50-2.84)	1.69 (1.16-2.47)	1.12 (0.60-2.12)	<.001
Generalized anxiety disorder^c^	450 (8.3)	31 (18.1)	2.45 (1.57-3.85)	16 (16.7)	2.22 (1.39-3.54)	4 (9.1)	1.11 (0.43-2.89)	<.001	NA^c^	NA^c^	NA^c^	NA^c^
Social phobia^c^	336 (6.3)	30 (17.7)	3.19 (2.15-4.73)	14 (14.9)	2.60 (1.46-4.64)	3 (7.0)	1.11 (0.34-3.63)	<.001	NA^c^	NA^c^	NA^c^	NA^c^
ADHD^c^	361 (6.7)	21 (13.0)	2.07 (1.32-3.26)	6 (7.0)	1.04 (0.48-2.23)	7 (14.9)	2.42 (1.13-5.19)	.001	NA^c^	NA^c^	NA^c^	NA^c^
Disability diagnosis												
None	5303 (88.1)	158 (75.6)	1 [Reference]	94 (80.3)	1 [Reference]	40 (78.4)	1 [Reference]	NA	1 [Reference]	1 [Reference]	1 [Reference]	NA
Any	716 (11.9)	51 (24.4)	2.39 (1.68-3.40)	23 (19.7)	1.56 (0.99-2.47)	11 (21.6)	2.04 (0.93-4.44)	<.001	2.10 (1.45-3.03)	1.43 (1.16-2.47)	1.99 (0.87-4.56)	<.001
Visual impairment	340 (6.0)	26 (14.1)	2.57 (1.65-4.00)	7 (7.2)	1.21 (0.58-2.52)	3 (7.0)	1.17 (0.36-3.83)	<.001	NA^d^	NA^d^	NA^d^	NA^d^
Autism	144 (2.6)	20 (11.2)	4.66 (2.78-7.82)	5 (5.3)	2.05 (0.87-7.81)	1 (2.4)	0.92 (0.13-6.45)	.001	NA^d^	NA^d^	NA^d^	NA^d^
Learning disability	125 (2.3)	5 (3.1)	1.34 (0.53-3.37)	3 (3.2)	1.41 (0.45-4.43)	3 (7.0)	3.18 (0.97-10.4)	.20	NA^d^	NA^d^	NA^d^	NA^d^

Abbreviations: ADHD, attention-deficit hyperactivity disorder; aOR, adjusted odds ratio; ICSEA, index of community socioeducational advantage; NA, not applicable; SES, socioeconomic status.

^a^ Cisgender respondents were the reference group for unadjusted and adjusted analyses.

^b^ Overall *P* values are from combined Wald test for all categories combined after estimation of multinomial logistic regression model.

^c^ These variables were not statistically significant at the overall or individual category levels in the multivariate model and are not included for reasons of parsimony.

^d^ These are the 3 most commonly reported diagnoses within the category. Percentages and comparisons vs no mental health diagnosis or no disability diagnosis and thus do not match prevalences reported in the text and eTable 1 in Supplement 1. These diagnoses were not considered for inclusion in the multivariate model because of small numbers and collinearity.

Compared with cisgender status, in both bivariable and multivariable models, preferring not to report gender identity was positively associated with attending school in a major city (aOR, 2.08 [95% CI, 1.10-3.95]), preferring not to report perceived family SES (aOR, 2.04 [95% CI, 1.32-3.15]), and having any mental health diagnosis (aOR, 1.69 [95% CI, 1.16-2.47]). The association between preferring not to report gender and living with no family members was not maintained in the multivariable model (Table 3). Before and after adjustment for other variables, missing gender identity was associated only with preferring not to report perceived family SES.

### Individual and School Characteristics by Sexuality Identity

Before and after adjustment for individual and school characteristics, compared with heterosexual identity, sexuality diversity was negatively associated with age (aOR per 1 year, 0.76 [95% CI, 0.63-0.91]) and positively associated with living with a single parent (aOR, 1.37 [95% CI, 1.06-1.75]), having any mental health diagnosis (aOR, 2.27 [95% CI, 1.89-2.73]), and having any disability diagnosis (aOR, 1.66 [95% CI, 1.37-2.00]) (Table 4). Gender diversity was positively associated with the 3 most common mental health and disability diagnoses in unadjusted analysis, but as with sexuality diversity, this could not be explored in multivariable modeling. Before and after adjustment, being unsure of sexuality was positively associated with speaking a language other than English in the home (aOR, 1.80 [95% CI, 1.27-2.57]). Before and after adjustment, preferring not to report sexuality was positively associated with attending a government school (1.62 [95% CI, 1.19-2.20]) and preferring not to report perceived family SES (aOR, 2.14 [95% CI, 1.58-2.91]). Univariate associations between preferring not to report sexuality identity and living with a single parent or with low school socioeducational advantage were not maintained after adjustment for other variables (Table 4). Before and after adjustment, missing responses to the sexuality identity item were positively associated with low perceived family SES (OR, 1.76 [95% CI, 1.23-2.51]) and preferring not to report family SES (OR, 1.49 [95% CI, 1.12-1.98]), and with living with a single parent (OR, 1.69 [95% CI, 1.22-2.35]). After adjustment, missing responses to the sexuality identity item were negatively associated with attending a government school. Positive associations between missing or uninterpretable responses to the sexuality identity item and mental health and disability diagnoses were not maintained after adjustment for other variables (Table 4). A multivariable multinomial regression model of sexuality identity including adjustment for gender identity is presented as a sensitivity analysis in eTable 2 in Supplement 1. Most estimates changed no more than 0.1 in these analyses.

**Table 4.** Unadjusted (Bivariate) and Adjusted (Multivariate) Multinomial Regressions of Sexuality Diversity on Individual and School Characteristics

Variable	Heterosexual, No. (%) (n = 4472)^a^	Sexuality diverse (n = 767)		Unsure (n = 559)		Prefer not to report (n = 296)		Missing (n = 294)		Overall *P* value^b^	aOR (95% CI)^a^				Overall *P *value^b^
		No. (%)	OR (95% CI)	No. (%)	OR (95% CI)	No. (%)	OR (95% CI)	No. (%)	OR (95% CI)		Sexuality-diverse (n = 767)	Unsure (n = 559)	Prefer not to report (n = 296)	Missing, (n = 294)	
Age, median (IQR) [range], per 1-y increase	13.9 (13.6-14.3) [10.7-17.5]	13.8 (13.5-14.2) [11.5-16.6]	0.78 (0.65-0.93)	13.9 (13.5-14.2) [11.4-16.9]	0.88 (0.74-1.05)	13.9 (13.5-14.2) [12.4-16.5]	0.94 (0.76-1.18)	13.9 (13.6-14.3) [12.4-16.2]	1.02 (0.82-1.25)	.07	0.76 (0.63-0.91)	0.89 (0.75-1.05)	0.94 (0.77-1.16)	1.01 (0.83-1.24)	.05
Gender identity															
Cisgender	4408 (98.6)	539 (70.3)	1 [Reference]	527 (94.3)	1 [Reference]	271 (91.5)	1 [Reference]	274 (93.2)	1 [Reference]	<.001	NA^c^	NA^c^	NA^c^	NA^c^	NA^c^
Gender-diverse	20 (0.4)	162 (21.1)	66.2 (38.2-114.8)	12 (2.1)	5.02 (2.01-12.53)	6 (2.0)	4.88 (2.01-11.86)	9 (3.1)	7.24 (2.80-18.73)		NA^c^	NA^c^	NA^c^	NA^c^	
Prefer not to report	12 (0.3)	62 (8.1)	42.3 (22.8-78.3)	14 (2.5)	9.76 (4.52-21.07)	15 (5.1)	20.3 (9.70-42.6)	6 (2.0)	8.04 (3.54-18.27)		NA^c^	NA^c^	NA^c^	NA^c^	
Missing or uninterpretable	32 (0.7)	4 (0.5)	1.02 (0.35-2.95)	6 (1.1)	1.57 (0.67-3.66)	4 (1.4)	2.03 (0.75-5.53)	5 (1.7)	2.51 (1.06-5.96)		NA^c^	NA^c^	NA^c^	NA^c^	
School type															
Government	2211 (49.4)	431 (56.2)	1.31 (0.95-1.82)	308 (55.1)	1.25 (0.95-1.66)	183 (61.8)	1.66 (1.23-2.23)	119 (40.7)	0.70 (0.43-1.12)	.01	1.26 (0.92-1.77)	1.21 (0.91-1.61)	1.62 (1.19-2.20)	0.63 (0.40-1.00)	.01
Nongovernment	2261 (50.6)	336 (43.8)	1 [Reference]	251 (44.9)	1 [Reference]	113 (38.2)	1 [Reference]	175 (59.5)	1 [Reference]		1 [Reference]	1 [Reference]	1 [Reference]	1 [Reference]	
School ICSEA															
1000 or less	1289 (28.8)	228 (29.7)	1.04 (0.74-1.47)	169 (30.2)	1.07 (0.80-1.44)	123 (41.6)	1.77 (1.27-2.42)	78 (26.5)	0.89 (0.52-1.52)	.002	NA^c^	NA^c^	NA^c^	NA^c^	NA^c^
1001 or greater	3183 (71.2)	539 (70.3)	1 [Reference]	390 (69.8)	1 [Reference]	173 (58.4)	1 [Reference]	216 (73.5)	1 [Reference]		NA^c^	NA^c^	NA^c^	NA^c^	
Location															
Major cities	3395 (75.9)	594 (77.4)	1.09 (0.80-1.49)	432 (77.3)	1.08 (0.82-1.41)	210 (70.9)	0.77 (0.58-1.03)	224 (76.2)	1.02 (0.57-1.81)	.27	NA^d^	NA^d^	NA^d^	NA^d^	NA^d^
Regional	1077 (24.1)	173 (22.6)	1 [Reference]	127 (22.7)	1 [Reference]	86 (29.1)	1 [Reference]	70 (23.8)	1 [Reference]		NA^d^	NA^d^	NA^d^	NA^d^	
Country of birth															
Australia	4102 (91.7)	694 (90.5)	1 [Reference]	500 (89.4)	1 [Reference]	276 (93.2)	1 [Reference]	270 (91.8)	1 [Reference]	.32	NA^d^	NA^d^	NA^d^	NA^d^	NA^d^
Other	370 (8.3)	73 (9.5)	1.17 (0.90-1.51)	59 (10.6)	1.31 (0.99-1.74)	20 (6.8)	0.80 (0.46-1.40)	24 (8.2)	0.99 (0.59-1.65)		NA^d^	NA^d^	NA^d^	NA^d^	
Language spoken at home															
English	4208 (94.1)	717 (93.5)	1 [Reference]	502 (89.8)	1 [Reference]	279 (94.3)	1 [Reference]	276 (93.9)	1 [Reference]	.007	1 [Reference]	1 [Reference]	1 [Reference]	1 [Reference]	.01
Other	263 (5.9)	50 (6.5)	1.12 (0.73-1.71)	57 (10.2)	1.82 (1.30-2.54)	17 (5.7)	0.97 (0.54-1.78)	18 (4.4)	1.04 (0.58-1.86)		1.17 (0.74-1.86)	1.80 (1.27-2.57)	0.90 (0.50-1.61)	1.11 (0.63-1.94)	
Perceived SES															
Medium/high	3504 (78.4)	600 (78.2)	1 [Reference]	443 (79.2)	1 [Reference]	199 (67.2)	1 [Reference]	203 (69.0)	1 [Reference]	<.001	1 [Reference]	1 [Reference]	1 [Reference]	1 [Reference]	<.001
Low	359 (8.0)	70 (9.1)	1.14 (0.85-1.52)	41 (7.3)	0.90 (0.64-1.27)	21 (7.1)	1.02 (0.59-1.79)	38 (12.9)	1.82 (1.27-2.64)		0.88 (0.66-1.18)	0.80 (0.57-1.13)	0.88 (0.50-1.53)	1.76 (1.23-2.51)	
Prefer not to report	609 (13.6)	97 (12.6)	0.93 (0.73-1.18)	75 (13.4)	0.97 (0.74-1.28)	76 (25.7)	2.20 (1.62-2.97)	53 (18.0)	1.50 (1.13-1.99)		0.89 (0.70-1.14)	0.95 (0.73-1.25)	2.14 (1.58-2.91)	1.49 (1.12-1.98)	
Family structure															
2 Parents	3582 (80.1)	563 (73.4)	1 [Reference]	429 (76.7)	1 [Reference]	219 (74.0)	1 [Reference]	216 (73.4)	1 [Reference]	<.001	1 [Reference]	1 [Reference]	1 [Reference]	1 [Reference]	.02
Blended/ stepfamily	430 (9.6)	87 (11.3)	1.29 (0.98-1.69)	67 (12.0)	1.30 (0.98-1.72)	36 (12.2)	1.37 (0.93-2.01)	28 (9.5)	1.08 (0.73-1.60)		1.10 (0.83-1.45)	1.28 (0.96-1.71)	1.24 (0.83-1.84)	1.06 (0.74-1.53)	
Single parent	420 (9.4)	106 (13.8)	1.61 (1.27-2.04)	61 (10.9)	1.21 (0.90-1.63)	37 (12.5)	1.44 (1.01-2.05)	45 (15.3)	1.78 (1.25-2.52)		1.37 (1.06-1.75)	1.20 (0.89-1.62)	1.34 (0.82-1.95)	1.69 (1.22-2.35)	
Other, no family	40 (0.9)	11 (1.4)	1.75 (0.82-3.75)	2 (0.4)	0.42 (0.12-1.47)	4 (1.4)	1.64 (0.55-4.83)	5 (1.7)	2.07 (0.81-5.28)		1.42 (0.64-3.14)	0.39 (0.11-1.39)	1.25 (0.42-3.77)	2.03 (0.81-5.10)	
Mental health diagnosis															
No diagnosis	3786 (84.7)	528 (68.8)	1 [Reference]	461 (82.5)	1 [Reference]	246 (83.1)	1 [Reference]	235 (79.9)	1 [Reference]	NA	1 [Reference]	1 [Reference]	1 [Reference]	1 [Reference]	NA
Any diagnosis	686 (15.3)	239 (31.2)	2.50 (2.10-2.98)	98 (17.5)	1.17 (0.94-1.47)	50 (16.9)	1.12 (0.78-1.62)	59 (20.1)	1.39 (1.04-1.84)	<.001	2.27 (1.89-2.73)	1.19 (0.95-1.49)	1.04 (0.72-1.52)	1.21 (0.90-1.62)	<.001
Generalized anxiety disorder	310 (7.6)	113 (17.6)	2.61 (2.05-3.33)	43 (8.5)	1.14 (0.81-1.59)	19 (7.2)	0.94 (0.54-1.66)	16 (6.4)	0.83 (0.51-1.34)	<.001	NA^e^	NA^e^	NA^e^	NA^e^	NA^e^
Social phobia	205 (5.1)	112 (17.5)	3.92 (3.10-4.94)	33 (6.7)	1.32 (0.95-1.84)	14 (5.4)	1.05 (0.58-1.91)	19 (7.5)	1.49 (0.96-2.32)	<.001	NA^e^	NA^e^	NA^e^	NA^e^	NA^e^
ADHD	237 (5.9)	73 (12.2)	2.21 (1.66-2.95)	41 (8.2)	1.42 (0.98-2.08)	14 (5.4)	0.94 (0.51-1.63)	30 (11.3)	2.04 (1.32-3.14)	<.001	NA^e^	NA^e^	NA^e^	NA^e^	NA^e^
Disability diagnosis															
No diagnosis	3973 (88.8)	615 (80.2)	1 [Reference]	494 (88.4)	1 [Reference]	260 (87.8)	1 [Reference]	249 (84.7)	1 [Reference]	NA	1 [Reference]	1 [Reference]	1 [Reference]	1 [Reference]	NA
Any diagnosis	499 (11.2)	152 (19.8)	1.96 (1.64-2.36)	65 (11.6)	1.05 (0.79-1.39)	36 (12.2)	1.10 (0.77-1.58)	45 (15.3)	1.44 (1.03-2.01)	<.001	1.66 (1.37-2.00)	1.02 (0.76-1.35)	1.09 (0.76-1.58)	1.37 (0.98-1.92)	<.001
Visual impairment	235 (5.6)	70 (10.2)	1.92 (1.46-2.53)	37 (7.0)	1.27 (0.88-1.82)	17 (6.1)	1.11 (0.67-1.81)	17 (6.4)	1.15 (0.69-1.92)	<.001	NA^e^	NA^e^	NA^e^	NA^e^	NA^e^
Autism	87 (2.1)	45 (6.8)	3.34 (2.33-4.80)	14 (2.8)	1.29 (0.75-2.22)	10 (3.7)	1.76 (0.90-3.42)	14 (5.3)	2.57 (1.38-4.77)	<.001	NA^e^	NA^e^	NA^e^	NA^e^	NA^e^
Learning disability	088 (2.2)	20 (3.1)	1.47 (0.93-2.31)	10 (2.0)	0.91 (0.49-1.70)	7 (2.6)	1.22 (0.58-2.57)	11 (4.2)	1.99 (0.97-4.10)	.16	NA^e^	NA^e^	NA^e^	NA^e^	NA^e^

Abbreviations: ADHD, attention-deficit hyperactivity disorder; aOR, adjusted odds ratio; NA, not applicable; SES, socioeconomic status.

^a^ Cisgender respondents were the reference group for unadjusted and adjusted analyses.

^b^ Overall *P *values are from combined Wald test for all categories combined after estimation of multinomial logistic regression model.

^c^ Gender identity is excluded from these models for interpretability. A model including gender identity is provided in eTable 2 in Supplement 1 as a sensitivity analysis.

^d^ These variables were not statistically significant at the overall or individual category levels in the multivariate model and are not included for reasons of parsimony.

^e^ These are the 3 most commonly reported diagnoses within the category for the cohort. Percentages and comparisons are compared with no mental health diagnosis or no disability diagnosis and thus do not match prevalences reported in the text and eTable 1 in Supplement 1. These diagnoses were not considered for inclusion in the multivariate model because of small numbers and collinearity.

## Discussion

To our knowledge, this cohort study presents the first population-representative data about sexual orientation and gender identity measured at the same time in young adolescents (median age, 13.9 years) and the only contemporary (2019-2022) population-representative information about Australian adolescents. We found that 3.3% (95% CI, 2.7%-3.9%) of respondents were gender diverse, higher than other recent representative school-based samples: the 2021 New Zealand National Youth Health and Well-being (What About Me?) Survey (years 9-13) found that 2.3% (95% CI, 1.9%-2.7%) of respondents were gender diverse^37^; the 2019 Minnesota Student Survey data (8th, 9th, and 11th grades), 1.4% of respondents^10^; and the 2017 New York, New York Youth Risk Behavior Survey (YRBS) (9th-12th grades),^11^ 1.5% of respondents. These studies did not report overall cohort age nor disaggregate gender identity by age, but the inclusion of students beyond year/grade 8 resulted in older populations than ours. Differences may also reflect the questions. Two-step gender ascertainment is current best practice^38,39^ and was used by the New Zealand survey but not the US surveys. The gender identity item we used was informed by the Australian Bureau of Statistics 2020 Standard,^40^ developed with extensive community consultation to standardize data collection. However, there was no unsure option for gender identity. This limited our capacity to understand gender fully. Very few participants in our sample (0.1%) provided uninterpretable response.

The other studies did provide unsure response options and found smaller proportions of gender-diverse adolescents. Given the stigma associated with gender diversity, it seems unlikely that adolescents who were unsure about their gender identity would, absent that response option, have chosen to report a diverse identity, hence explaining the higher prevalence in our sample. The Finland School Health Promotion Survey^41^ (grade 8 to postsecondary and vocational, age ≤21 years) used a 2-step method, without unsure or prefer not to say categories and then removed participants with implausible responses to age, height, body mass index, or disability status (3.5%). Before this elimination, 0.7% of respondents identified as transgender and 4.2% as nonbinary or other gender, and after, 0.6% and 3.3% of respondents, respectively, slightly higher than our overall prevalence of gender diversity. We did not assess overall plausibility of responses but uninterpretable responses to sexuality and gender items in a separate category, which did not contribute toward our prevalence of gender diversity. Taken together, this suggests that rather than the absence of an unsure category driving higher prevalences of gender diversity in our study, the lower proportion of gender diversity in the other studies reflects older participant ages, the slightly earlier era of some of the data collection, or cultural differences. While privacy may also be a consideration, similar to the YRBS, the Future Proofing Study asked students to sit at a distance if possible and complete surveys without consultation.

We found that a total of 12.0% of our sample was sexuality diverse, with 1.6% identifying as gay or lesbian, 6.5% identifying as bisexual, 1.9% identifying as pansexual, 1.0% identifying as asexual, and 1.0% using another term. Data from the Minnesota Survey^10^ found that 9.4% of respondents were sexuality diverse, less than our estimate. The 2019 National YRBS^42^ (9th-12th grades) found that 2.5% of respondents were gay or lesbian and 8.7% were bisexual, and the New York YRBS^11^ found that 3.1% of respondents were gay or lesbian and 8.0% of respondents were bisexual, both of which were higher than our estimates, possibly because of the presence of additional sexuality response options in our data collection. In the 2022 UK Census, among participants aged 16 to 24 years, 2.8% identified as gay or lesbian, 6.4% as bisexual, and 1.9% as another diverse identity, with 11.1% overall identifying as sexuality diverse.^15^ The 2023 British Columbia Adolescent Health Study^43^ (7th-12th grades) found a much higher proportion of sexuality-diverse young people, at approximately 21%, including 2% gay or lesbian, 10% bisexual or pansexual, 2% asexual, 6% mostly straight, and less than 1% something else. Some of this difference may be because of the mostly straight category—it is possible that some participants who identified as heterosexual or straight in our study would have counted themselves as mostly straight, given the option.

Again, some discrepancies in prevalence may be due to differences in ascertainment. There is discussion about how best to collect SGD identity,^40,44,45,46,47,48^ with evidence in adults that providing more options, including do not know or unsure, decreases nonresponse.^49,50^ In adults, answering do not know or unsure to, or refusing, sexual orientation items is associated with demographic and contextual variables^51,52,53^ and with adverse health and social outcomes (higher likelihood of substance use^18,19^ and violence,^19,25^ and poorer sleep,^28^ cardiovascular disease,^29^ and poorer mental health^30^). Individuals who are uncertain about or uncomfortable with disclosing their sexuality or gender identities may still be subject to stigmatization, including internalized, and discrimination. These uncertainties might also limit an individual’s sense of belonging to either cisgender heterosexual or SGD communities, with poorer outcomes by virtue of less support and connection.^54^ Uncertainty and questioning are likely different in adulthood than in adolescence, as adolescence is a time of identity development. However, some evidence suggests that, as in adults, adolescents who report being unsure of their sexuality experience adverse health and social outcomes, including violence and victimization,^10,12,20,26,27,55^ substance use,^20,21,22,23,24,55,56^ and poor mental health,^12,20,21,22,55^ albeit less commonly than do sexual and gender minority adolescents.

We also found that 1.8% of respondents preferred not to report their gender, similar to the New York YRBS (1.9%) as reported by Travers et al.^11^ While that study provided the option, “I do not know what this question is asking,” reported by 3.1%, Travers et al^11^ used a direct rather than 2-step approach, asking, “Some people describe themselves as transgender when their sex at birth does not match the way they think or feel about their gender. Are you transgender?” without defining *gender*. Respondents may not have understood what “sex at birth” or “gender” meant in this context.

While there were significant associations between individual and school characteristics and uncertain or evasive answers to gender and sexuality identity items, patterns of demographic characteristics differed, suggesting that they are interpreted as different concepts by respondents. Providing prefer not to report and unsure for gender and sexuality identity measures may help increase response rates in younger adolescents and possibly identify disparity. There was no readily apparent pattern of association between being economically or otherwise socially vulnerable and preferring not to report gender or sexuality identities. Qualitative research will deepen our understanding of the meaning and significance of these responses to young participants. This is important because of the associations seen with adverse health and social outcomes in adolescents and adults. We also need longitudinal studies, like the Future Proofing Study, to understand how these responses change as young people develop their identities over time.

Even in data limited to a single school year, SGD were associated with younger age, consistent with existing literature.^13,14,15,16,17^ As the youngest adolescents were the most likely to identify as SGD, considering the increased risk for stigma, discrimination, and violence, there is an urgent need for supportive interventions in schools, health care settings, and communities in this age group. The strong association between gender diversity and sexuality diversity and the well-recognized associations with mental health and disability diagnoses only increase this urgency.

### Limitations

This study has some limitations. While this study is broadly representative of the Australian population,^31^ limitations include that it took an opt-in approach requiring both parental and participant consent, it is not a probabilistic sample and, for logistical reasons, it overrepresents New South Wales schools vs schools in other Australian jurisdictions. Furthermore, surveys were completed on personal devices at school. While every effort was made to ensure privacy and space during survey completion, some students may have felt a lack of privacy, which may have impacted their responses. There are also limitations around assessment of diversity. Consistent with Australian Bureau of Statistics standards,^57^ cultural and linguistic diversity were assessed using language spoken at home and place of birth. However, although Indigenous status was collected at baseline, any disaggregation of the data of Indigenous peoples in Australia must be supported by the local Indigenous human research ethics committees, with close engagement and leadership with the communities in which the research has been conducted prior to initiation of the research. Thus, this analysis was not undertaken. The quality of self-reported data may vary, and in particular, perceived socioeconomic status, mental health diagnosis, and disability diagnosis may be less reliable than more direct measures. Additionally, like most general population surveys, this cohort has small absolute numbers of SGD young people, which limits our ability to compare subgroups.

## Conclusions

The findings of this cohort study previous studies reporting that, similar to adults, young adolescents can have firmly established views of their gender and sexuality, even at ages when many have not yet initiated romantic or sexual relationships.^58,59^ While social science research has identified gender and sexuality as identities established in childhood,^48,60,61^ this has not been documented or widely accepted in population health or health care. Our observations support recommendations for child health policies that promote inclusion and support of diverse gender identities and sexualities from a young age to mitigate the deleterious impact of minority stress and internalized transphobia and homophobia. Future research with younger adolescents, particularly longitudinal and population-based studies, should include items about sexuality and gender identities, and we should conduct ongoing qualitative research with community to ensure these items reflect lived experience of sexuality and gender diversity.

## Notes

Supplement 1. **eTable 1.** Demographics **eTable 2.** Multivariable (Adjusted) Multinomial Regressions of Sexuality Diversity on Individual and School Characteristics, Including Gender Diversity **eBox 1.** Questions About Sexuality and Gender, Response Options, and Optional Explainer Text **eBox 2.** Definitions of Gender and Sexuality Related Terms, Including Identities Provided by Study Participants

Supplement 2.
Data Sharing Statement
