# Supplementary material for: Sexuality and Gender Diversity Among Adolescents in Australia, 2019-2021
Source: JAMA Netw Open. 2024 Oct 28;7(10):e2444187. doi: 10.1001/jamanetworkopen.2024.44187 (PMC11581561; doi:10.1001/jamanetworkopen.2024.44187)
Supplement: Supplement 1. — eTable 1. Demographics eTable 2. Multivariable (Adjusted) Multinomial Regressions of Sexuality Diversity on Individual and School Characteristics, Including Gender Diversity eBox 1. Questions About Sexuality and Gender, Response Options, and Optional Explainer Text eBox 2. Definitions of Gender and Sexuality Related Terms, Including Identities Provided by Study Participants [file jamanetwopen-e2444187-s001.pdf]

Supplemental Online Content

Marino JL, Werner-Seidler A, Maston K, et al. Sexuality and gender diversity among adolescents in Australia, 2019-2021. *JAMA Netw Open*. 2024;7(10):e2444187. doi:10.1001/jamanetworkopen.2024.44187

eTable 1. Demographics

eTable 2. Multivariable (Adjusted) Multinomial Regressions of Sexuality Diversity on Individual and School Characteristics, Including Gender Diversity

eBox 1. Questions About Sexuality and Gender, Response Options, and Optional Explainer Text

eBox 2. Definitions of Gender and Sexuality Related Terms, Including Identities Provided by Study Participants

This supplemental material has been provided by the authors to give readers additional information about their work.

© 2024 Marino JL et al. *JAMA Network Open.*

eTable 1: Demographics

Characteristic	N (%, 95%CI)
Location	
Major city	4855 (76.0, 67.1-83.1)
Inner regional	1409 (22.1, 15.2-30.9)
Outer regional	124 (1.9, 0.9-4.2)
Country of birth	
Australia	5842 (91.5, 89.9-92.8)
Other	546 (8.5, 7.2-10.1)
Language spoken at home	
English	5982 (93.7, 91.6-95.2)
Other	405 (6.3, 4.8-8.4)
Indigenous status	
Not Aboriginal or Torres Strait Islander	5899 (92.3, 90.9-93.5)
Aboriginal or Torres Strait Islander	338 (5.3, 4.3-6.5)
Prefer not to say	151 (2.4, 2.0-2.8)
Perceived SES	
Not at all well-off	112 (1.8, 1.4-2.2)
Not particularly well-off	417 (6.5, 5.7-7.3)
Fairly well-off	2083 (32.6, 31.1-34.1)
Rather well-off	2016 (31.6, 29.8-33.3)
Very well-off	850 (13.3, 12.3-14.4)
Prefers not to say	910 (14.2, 12.9-15.6)
School sector	
Government	3252 (50.9, 40.3-61.4)
Non-government	3136 (49.1, 38.6-59.7)
School community socio-educational advantage ICSEA	
<=1001	1887 (29.5, 21.5-39.1)
=>1000	4501 (70.5, 60.9-78.5)
Family structure / household makeup	
Two parents	5009 (78.4, 76.5-80.2)
Blended/ stepfamily	648 (10.1, 9.0-11.4)
Single parent	669 (10.5, 9.5-11.5)
Other family members	55 (0.9, 6.4-11.5)
No family members	7 (0.1, 0.05-0.2)
Mental health diagnosis	Does not sum to 100%
Major depression	239 (3.7, 3.2-4.3)
Social anxiety disorder	383 (6.0, 5.3-6.8)
Generalised anxiety disorder	501 (7.8, 7.0-8.8)
Obsessive-compulsive disorder	85 (1.3, 1.0-1.7)
Panic disorder	120 (1.9, 1.5-2.3)
Separation anxiety	97 (1.5, 1.2-1.9)
Alcohol use disorder	8 (0.1, 0.05-0.3)
Substance use disorder	10 (0.2, 0.09-0.3)
ADHD	395 (6.2, 5.4-7.1)
PTSD	88 (1.4, 1.1-1.7)
Schizophrenia/psychosis	16 (0.3, 0.2-0.4)
None of the above	5253 (82.2, 80.8-83.6)
Any of the above	1135 (17.7, 16.4-19.2)
Disability diagnosis	Does not sum to 100%
Autism spectrum disorders	170 (2.7, 2.2-3.2)
Intellectual disability	29 (0.5, 0.3-0.7)
Specific learning disability	136 (2.1, 1.8-2.6)
Tourette or tic disorder	41 (0.6, 0.5-0.9)
Cerebral palsy	9 (0.1, 0.07-0.3)
Acquired brain injury	16 (0.3, 0.2-0.4)
Other neurological disability	57 (0.9, 0.7-1.2)
Hearing impairment	90 (1.4, 1.1-1.7)
Visual impairment	376 (5.9, 5.2-6.7)
None of the above	5591 (87.5, 86.5-88.4)
Any of the above	797 (12.5, 11.6-13.5)

© 2024 Marino JL et al. *JAMA Network Open.*

eTable 2: Multivariable (adjusted) multinomial regressions of sexuality diversity on individual and school characteristics, including gender diversity

**Characteristic**	**Heterosexual N=4472 N (%) OR (95%CI)**	**Diverse sexual identity N=767 N (%) OR (95%CI)**	**Prefers not to say N=559 N (%) OR (95%CI)**	**Unsure N=296 N (%) OR (95%CI)**	**Missing N=294 N (%) OR (95%CI)**
Age	Overall p=0.26				
	Reference	**0.82 (0.68-0.99)**	0.89 (0.75-1.05)	0.94 (0.76-1.16)	1.02 (0.83-1.25)
Gender identity	Overall p<0.0001				
Cisgender	Reference	Reference	Reference	Reference	Reference
Gender diverse	Reference	**60.03 (33.73-106.86)**	**4.80 (1.92-11.99)**	**4.25 (1.73-10.44)**	**7.01 (2.74-17.91)**
Prefers not to say	Reference	**42.12 (23.42-75.75)**	**9.66 (4.52-20.66)**	**17.61 (8.34-37.16)**	**7.11 (3.16-15.97)**
Missing	Reference	0.97 (0.33-2.84)	1.52 (0.65-3.51)	1.86 (0.64-4.98)	2.20 (0.94-5.17)
School type	Overall p=0.010				
Government	Reference	1.15 (0.83-1.60)	1.20 (0.91-1.59)	**1.61 (1.18-2.18)**	**0.63 (0.40-0.99)**
Non-government	Reference	Reference	Reference	Reference	Reference
Language spoken at home	Overall p=0.011				
English	Reference	Reference	Reference	Reference	Reference
Other	Reference	1.07 (0.67-1.71)	**1.78 (1.26-2.53)**	0.87 (0.48-1.59)	1.08 (0.62-1.89)
Perceived SES (well-off)	Overall p<0.0001				
Medium/high	Reference	Reference	Reference	Reference	Reference
Low	Reference	0.86 (0.63-1.17)	0.80 (0.57-1.12)	0.87 (0.50-1.54)	**1.74 (1.22-2.48)**
Prefers not to say	Reference	0.75 (0.57-1.00)	0.92 (0.70-1.21)	**2.03 (1.49-2.76)**	**1.44 (1.09-1.92)**
Family structure	Overall p=0.035				
Two parents	Reference	Reference	Reference	Reference	Reference
Blended/ stepfamily	Reference	0.98 (0.68-1.41)	1.26 (0.95-1.68)	1.20 (0.80-1.78)	1.03 (0.72-1.48)
Single parent	Reference	**1.32 (1.00-1.75)**	1.19 (0.88-1.60)	1.33 (0.91-1.93)	**1.67 (1.20-2.33)**
Other, no family	Reference	1.07 (0.47-2.49)	0.36 (0.10-1.31)	1.12 (0.37-3.40)	1.86 (0.74-4.65)
Mental health diagnosis	Overall p<0.0001				
None	Reference	Reference	Reference	Reference	Reference
Any	Reference	**2.17 (1.75-2.69)**	1.19 (0.95-1.49)	1.04 (0.72-1.51)	1.20 (0.89-1.61)
Disability diagnosis	Overall p=0.0046				
None	Reference	Reference	Reference	Reference	Reference
Any	Reference	**1.48 (1.18-1.85)**	1.00 (0.75-1.34)	1.07 (0.74-1.55)	1.34 (0.96-1.87)

© 2024 Marino JL et al. *JAMA Network Open.*

eBox 1 – Questions about sexuality and gender, response options, and optional explainer text

Question	Response options	Explainer text[a]
What sex were you assigned at birth, on your original birth certificate?	Female	This question is asking whether you were a male or female when you were born.
	Male	
	Not sure	
	I prefer not to say	
	Another term (specify)	
What is your current *gender identity*?	Female	This is whether you feel that you are a male or female, which may be different to sex recorded at birth or what is indicated on legal documents.
	Male	
	Non-binary	
	Other (specify)	
	I prefer not to say	
Do you consider your sexual orientation to be	*Heterosexual or straight*	This means attracted to a member of the opposite sex
	Gay or lesbian	This means attracted to a member of the same sex
	Bisexual	This means attracted to members of both the same and different sexes (e.g., males and females)
	Pansexual	This means attracted to people regardless of their gender. That is, it doesn't matter to you if a person is male or female because this is irrelevant to you
	Asexual	This means not attracted to anyone
	Other (specify)	(no explainer text)
	Not sure	(no explainer text)
	Prefer not to say	(no explainer text)
People are different in their sexual attraction to other people. Which best describes your feelings?	Only attracted to females	(no explainer text)
	Mostly attracted to females	
	Equally attracted to females and males	
	Mostly attracted to males	
	Only attracted to males	
	Other	
	Not sure	
	Prefer not to say	

a. Text appeared if the participant hovered the cursor over the italic text in the Question or Response options.

© 2024 Marino JL et al. *JAMA Network Open.*

eBox 2: Definitions of gender and sexuality related terms, including identities provided by study participants[1-8]

Term[a]	Definition
Cisgender	A person whose gender is consonant with the sex recorded for them at birth (cf transgender)
Gender	The range of characteristics that a culture identifies as masculine or feminine
Gender expression	A person's outward gender presentation and behaviour
Gender identity	A person's internal view of their gender
Transgender	A person whose gender is not consonant with the sex recorded for them at birth (cf cisgender)
**Gender identities provided by participants**	
Agender	A person who experiences very little or no connection to gender (binary or nonbinary) as a primary component of identity
Bigender	A person who experiences two distinct genders, at the same time or in flux; these genders may include man/boy or woman/girl
Butterfly	A non-binary gender which feels small and moves about like a butterfly
Demigirl	A non-binary gender in which a person identifies partially with being a woman/girl
Demiboy	A non-binary gender in which a person identifies partially with being a man/boy
Genderfae	A person whose gender experience is fluid but does not encompass male or masculine genders
Genderfluid	A person whose gender identity and/or presentation is not fixed (changes amongst genders, which may include no gender, over time)
Genderqueer	An umbrella term including all people whose gender identity and/or presentation do not conform to fixed binary gender norms
Inexgender or implagender	A person who is never satisfied with their gender because of self-doubt, and who feels the need to continue to search for a truer gender
**Sexuality identities provided by participants**	
Abrosexual	A person with a fluid or rapidly changing sexuality that fluctuates amongst sexualities
Bi-curious	A person who is interested in exploring their attraction to people of two or more genders
Biromantic	A person who experiences romantic attraction to people of two or more genders
Demiromantic	A person who experiences romantic attraction only after forming a close emotional (often sexual) relationship
Demisexual	A person who experiences sexual attraction only after forming a close emotional (often romantic) relationship
Greyromantic	A partially aromantic person – a person who only rarely experiences romantic attraction, or who experiences romantic attraction but does not desire romantic relationships
Greysexual	A partially asexual person – a person who only rarely experiences sexual attraction, or who experiences sexual attraction but does not desire romantic relationships
Heteroflexible	A person who identifies as straight but sometimes experiences attraction to people of their own gender
Lithoromantic	A person who experiences romantic attraction but does not need or does not desire those feelings to be reciprocated, or whose romantic attraction fades when reciprocated
Neptunic	A person who is attracted to women, feminine non-binary people, and neutral non-binary people; or, a person who is attracted to people of any except masculine-aligned genders
Omniromantic	A person who is romantically attracted to people of any gender(s), with gender playing a role in the attraction
Omnisexual	A person whose sexual, romantic or physical attraction is people of any gender(s), with gender playing a role in the attraction
Panromantic	A person who is romantically attracted to people of any gender(s), for whom gender plays no role in the attraction
Polysexual	A person who is sexually attracted to people of many but not necessarily all genders
Queer	An umbrella term including all people whose sexuality or gender does not conform to cisgender heterosexual
Trixic	A non-binary person who is attracted to women

a. Please note that definitions may not reflect the participant's experience exactly and may shift over time. As some identities are stigmatized, some terms may be considered offensive when used by people who do not share the identity.

1. Anonymous. Fandom LGBTQIA+ Wiki Main Page. Accessed August 12, 2024. https://lgbtqia.fandom.com/wiki/LGBTQIA%2B_Wiki
2. Human Rights Campaign Foundation. Glossary of Terms. Updated 31/5/2023. Accessed August 12, 2024. https://www.hrc.org/resources/glossary-of-terms
3. Safe Zone Project. LGBTQ+ VOCABULARY GLOSSARY OF TERMS. Accessed August 12, 2024. https://thesafezoneproject.com/resources/vocabulary/

© 2024 Marino JL et al. *JAMA Network Open.*

4. PFLAG. PFLAG NATIONAL GLOSSARY. Accessed August 12, 2024. https://pflag.org/glossary/
5. Project IGB. Glossary. Accessed August 12, 2024. https://itgetsbetter.org/glossary/
6. Farrugia C. LGBTIQA+ glossary of common terms. Melbourne, Australia: Australian Institute of Family Studies; 2022.
7. (AVEN) AVaEN. AVENWiki Lexicon. Accessed August 12, 2024. http://wiki.asexuality.org/Lexicon
8. Davies C, Robinson KH, Metcalf A, et al. Australians of diverse sexual orientations and gender identities. In: Dune T, McLeod K, Williams R, eds. *Culture, Diversity and Health in Australia: Towards Culturally Safe Health Care*. Routledge; 2021.

© 2024 Marino JL et al. *JAMA Network Open.*
