# Supplementary material for: Sexuality and Gender Diversity Among Adolescents in Australia, 2019-2021
Source: JAMA Netw Open. 2024 Oct 28;7(10):e2444187. doi: 10.1001/jamanetworkopen.2024.44187 (PMC11581561; doi:10.1001/jamanetworkopen.2024.44187)
Supplement: Supplement 2. — Data Sharing Statement [file jamanetwopen-e2444187-s002.pdf]

Data Sharing Statement

Marino. Sexuality and Gender Diversity Among Adolescents in Australia, 2019-2021. *JAMA Netw Open*. Published October 28, 2024. doi:10.1001/jamanetworkopen.2024.44187

Data
Data available: No

Additional Information
Explanation for why data not available: Due to privacy/ethical restrictions, the data are not publicly available but are available on request to the Future Proofing Study Committee, Black Dog Institute.
